# Incidence and Outcomes of Laryngeal Complications Following Adult Cardiac Surgery: A National Analysis

**DOI:** 10.1007/s00455-021-10377-2

**Published:** 2021-10-21

**Authors:** Arjun Verma, Joseph Hadaya, Zachary Tran, Vishal Dobaria, Josef Madrigal, Yu Xia, Yas Sanaiha, Abie H. Mendelsohn, Peyman Benharash

**Affiliations:** 1 Cardiovascular Outcomes Research Laboratories (CORELAB), Division of Cardiac Surgery, David Geffen School of Medicine at UCLA, Los Angeles, CA USA; 2 Division of Laryngology, Department of Head and Neck Surgery, David Geffen School of Medicine at UCLA, Los Angeles, CA USA

**Keywords:** Laryngeal complications, Cardiac surgery, Dysphagia, Vocal fold paralysis, Nationwide Readmissions Database

## Abstract

Laryngeal complications (LCs) following cardiac operations contribute to increased morbidity and resource utilization. Using a nationally representative cohort of cardiac surgical patients, we characterized the incidence of LC as well as its associated clinical and financial outcomes. All adults undergoing coronary artery bypass grafting and/or valvular operations were identified using the 2010–2017 Nationwide Readmissions Database. International Classification of Diseases 9th and 10th Revision diagnosis codes were used to identify LC. Trends were analyzed using a rank-based, non-parametric test (nptrend). Multivariable linear and logistic regressions were used to evaluate risk factors for LC, and its impact on mortality, complications, resource use and 30-day non-elective readmissions. Of an estimated 2,319,628 patients, 1.7% were diagnosed with perioperative LC, with rising incidence from 1.5% in 2010 to 1.8% in 2017 (nptrend < 0.001). After adjustment, female sex [adjusted odds ratio 1.08, 95% confidence interval (CI) 1.04–1.12], advancing age, and multi-valve procedures (1.51, 95% CI 1.36–1.67, reference: isolated CABG) were associated with increased odds of LC. Despite no risk-adjusted effect on mortality, LC was associated with increased odds of pneumonia (2.88, 95% CI 2.72–3.04), tracheostomy (4.84, 95% CI 4.44–5.26), and readmission (1.32, 95% CI 1.26–1.39). In addition, LC was associated with a 7.7-day increment (95% CI 7.4–8.0) in hospitalization duration and $24,200 (95% CI 23,000–25,400) in attributable costs. The present study found LC to be associated with increased perioperative sequelae and resource utilization. The development and application of active screening protocols for post-surgical LC are warranted to increase early detection and reduce associated morbidity.

**Supplementary Information:**

The online version contains supplementary material available at 10.1007/s00455-021-10377-2.

## Introduction

Postoperative laryngeal complications (LCs) resulting in poor voice and swallow function are an increasingly recognized sequelae of several surgical procedures [1–3]. Generally attributable to laryngeal injury, dysphagia after cardiac surgery is estimated to occur between 2 and 16% of patients, with risk factors including congestive heart failure, diabetes mellitus, and advanced age [4–7]. Postoperative LCs result from either direct injury to the laryngeal structures, recurrent laryngeal or vagus nerves from endotracheal intubation, transesophageal echocardiography (TEE), and/or direct impact of surgical dissection [3, 8–10]. Widespread use of intraoperative TEE and prolonged operative length have also been suggested to contribute to the growing prevalence of LCs in this select group [3, 7, 10].

While some patients with laryngeal injury exhibit weak phonation, vocal fold hypomobility is not reliably identified via auditory perception of voice. Moreover, many patients demonstrate dysphonia with completely normal vocal fold motion. The term “silent aspiration” generally refers to patients who aspirate saliva or oral nutritional intake without demonstrating discomfort or producing a cough. Such “asymptomatic” aspiration of contents into the respiratory tract increases the risk of pneumonia, respiratory failure and ensuing systemic illness [5, 11, 12]. Following cardiac surgery, dysphagia has been associated with higher rates of aspiration pneumonia, cardiovascular complications, as well as postoperative tracheostomy and feeding tube utilization [3, 11, 13, 14]. Furthermore, patients with LC have been shown to experience delayed hospital discharge, greater in-hospital resource use, and reduced quality of life [12, 15, 16]. Thus, several clinical methods have been devised to screen and diagnose these conditions with fiberoptic endoscopic evaluation of swallowing (FEES) being the most common, as it can assess both swallow and vocal fold mobility in a reliable fashion [17, 18]. However, a universally adopted screening tool for post-surgical dysphonia and dysphagia remains lacking [19].

Risk factors and outcomes associated with laryngeal injury following cardiac operations have not yet been characterized at the national level. The present study utilized a nationally representative database to identify factors associated with LC and to evaluate its impact on clinical outcomes and resource utilization. We hypothesized LCs to be associated with increased mortality, complications, resource use, and non-elective readmissions.

## Materials and Methods

### Data Source and Cohort Definitions

The 2010–2017 Nationwide Readmissions Database (NRD), maintained by the Agency for Healthcare Research and Quality, served as the data source for the present study. The NRD is an all-payer national database that samples approximately 17 million inpatient hospitalization discharges annually, providing accurate estimates for 58.7% of all US hospitalizations. All adults (age ≥ 18) undergoing coronary artery bypass grafting (CABG) and/or valve operations were identified using *International Classification of Disease, 9–10th Revisions* procedure codes (ICD-9, ICD-10) [20]. Patients undergoing heart transplantation, implantation of durable ventricular assist devices, or transcatheter interventions, as well as those with missing data were excluded (1.2%). Within this cohort, LC was identified with ICD-9/10 diagnosis codes for conditions including vocal fold paralysis/paresis, dysphagia, dysphonia, aphagia, and aphonia (Supplemental Table 1). Patients without LC codes were considered absent of post-surgical laryngeal injury (nLC).

### Variable Definitions and Outcomes

Patient and hospital characteristics, including age, sex, hospital teaching status, size and region, were defined according to the HCUP data dictionary [21]. The van Walraven modification of the Elixhauser Comorbidity Index was used to quantify the extent of chronic conditions [22]. Specific comorbidities and complications were defined using ICD-9/10 diagnosis codes and in accordance with the NRD Data Dictionary [21]. End-stage renal disease (ESRD) was defined using previously validated diagnosis codes [23]. Hospitals were stratified into low- (LVH), medium- (MVH), and high-volume (HVH) tertiles based on annual institutional caseload of CABG and valvular operations. As hospitals are not tracked across years in the NRD, volume cutoffs were determined at the 33rd and 67th percentiles of volume for each year. Complications of interest included neurologic (cerebral infarction, transient ischemic attack), cardiac (ventricular tachycardia/fibrillation, cardiac arrest, tamponade), thrombotic (deep vein thrombosis, pulmonary embolism), and infectious (urinary tract infection, bacterial infection, clostridium difficile colitis, wound disruption, postoperative abscess, mediastinitis) [20, 24]. Tracheostomy, prolonged mechanical ventilation (> 96 h), and reintubation were among interventions assessed and were identified using appropriate ICD-9/10 procedure codes. Hospitalization costs were derived by application of hospital specific cost-to-charge ratios to overall charges and inflation adjusted to the 2017 Personal Health Care Index [25].

The primary outcome of the present study was the diagnosis of LCs. In-hospital mortality, complications, length of stay (LOS), hospitalization costs, discharge disposition, and 30-day non-elective readmissions were also assessed.

### Statistical Analysis

Temporal trends were analyzed using a rank-based, non-parametric test by Cuzick (nptrend) [26]. Categorical variables are reported as proportions (%) and compared using Pearson’s *χ*^2^ test. Continuous variables are reported as medians with interquartile range (IQR) and compared with the Mann–Whitney *U* test. Multivariable logistic regression models were developed to identify patient, hospital, and operative characteristics independently associated with LC. Additional models were developed to evaluate the association of laryngeal injuries with clinical outcomes of interest as well as LOS and hospitalization costs. Regression outcomes are reported as adjusted odds ratios (AORs) for dichotomous variables and beta coefficients (*β*) for continuous, both with 95% confidence intervals (95% CIs). Covariates were selected by applying stepwise backward elimination, with retention of clinically relevant independent variables. Model discrimination and overfitting were evaluated using receiver operating characteristics as well as the Akaike and Bayesian information criteria. The cumulative risk of 30-day, non-elective readmissions was evaluated using a Royston–Parmar flexible parametric model to adjust for differences in patient and hospital characteristics between groups. Unlike Cox proportional hazard models, Royston–Parmar survival analyses allow for varying hazards over time [27].

Statistical analysis was performed using Stata 16.0 (StataCorp, College Station, TX) software. Statistical significance was set at *α* < 0.05. This study was deemed exempt from full review by the Institutional Review Board at the University of California, Los Angeles.

## Results

Of an estimated 2,319,628 cardiac surgical patients, 39,688 (1.7%) were diagnosed with at least one postoperative LC. The annual incidence of LC continued to rise over the last decade from 1.5% in 2010 to 1.8% in 2017 (nptrend < 0.001). Patients in the LC cohort were more commonly female, older (72 years [IQR 63–79] vs. 67 [IQR 59–74], *p* < 0.001), insured by Medicare (70.8 vs. 56.7%, *p* < 0.001) and had a higher Elixhauser comorbidity index (4 [IQR 2–5] vs. 3 [IQR 2–5], *p* < 0.001). Specifically, rates of congestive heart failure, coagulopathy, and liver disease were increased among those with LC (Table 1). Compared to others, patients who developed LC less frequently underwent isolated CABG (52.1 vs. 61.5%, *p* < 0.001). Moreover, patients with LC were more frequently treated at HVH and LVH, compared to MVH (Table 1).

**Table 1:** Patient demographics, comorbidities, and clinical characteristics stratified by laryngeal complications

Parameter	LC (*n* = 39,688)	nLC (*n* = 2,279,940)	*p*-value
Age (years, median, IQR)	72 [63–79]	67 [59–74]	< 0.001
Female (%)	35.1	30.8	< 0.001
Elixhauser index (median, IQR)	4 [2–5]	3 [2–5]	< 0.001
Elective admission	49.2	54.0	< 0.001
Income quartile (percentile) (%)	< 0.001		
0th–25th	26.2	27.1	
26th–50th	25.7	26.9	
51st–75th	25.8	25.1	
76th–100th	22.3	20.9	
Insurance type (%)	< 0.001		
Private	18.7	30.3	
Medicare	70.8	56.7	
Medicaid	6.0	6.7	
Other payer	4.5	6.3	
Operation type (%)			< 0.001
Isolated CABG	52.1	61.5	
Isolated valve	26.4	23.8	
CABG + valve	17.4	11.9	
Multiple valve	4.0	2.8	
Comorbidities (%)			
Congestive heart failure	38.2	31.0	< 0.001
Coronary artery disease	63.3	71.3	< 0.001
Valve disorder	46.9	41.7	< 0.001
Chronic lung disease	23.9	22.2	0.003
Diabetes	27.5	34.2	< 0.001
End-stage renal disease	3.3	3.1	0.10
Liver disease	3.3	2.8	0.001
Coagulopathy	24.3	20.0	< 0.001
Endocarditis	2.8	2.2	< 0.001
Hospital volume			< 0.001
Low	3.1	2.1	
Medium	21.5	23.2	
High	75.4	74.8	
Teaching status	74.3	71.1	< 0.001

*CABG* coronary artery bypass grafting, *LC* laryngeal complications, *nLC* no diagnosed laryngeal complications

**Fig. 1:**
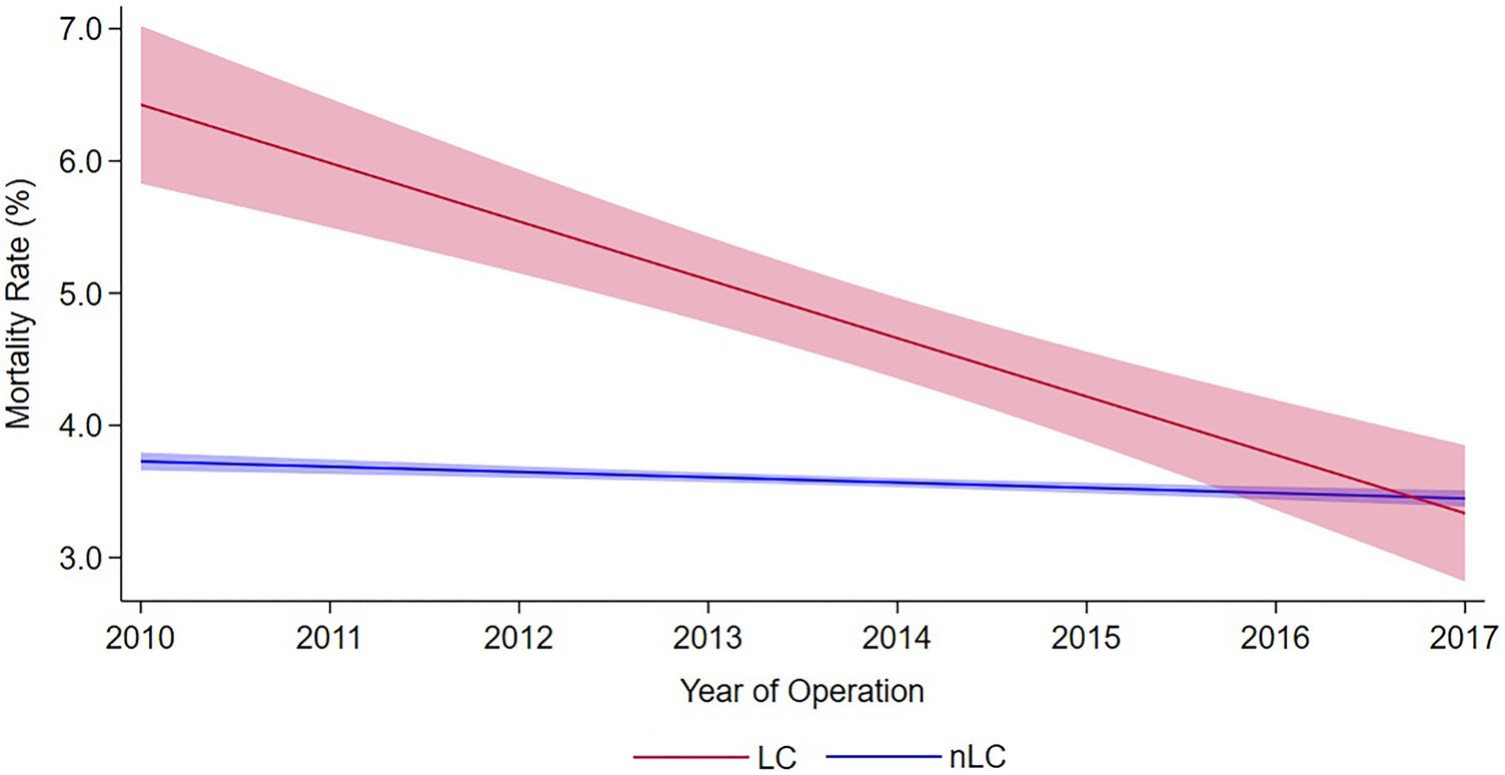
Temporal trends with 95% confidence intervals for in-hospital mortality for cardiac surgical patients classified by the presence of laryngeal complications. *LC* laryngeal complications, *nLC* no diagnosed laryngeal complications

Over the study period, unadjusted mortality rates significantly declined in both groups, with a more pronounced decrease among patients with LC (Fig. 1). Nonetheless, patients with LC demonstrated higher unadjusted rates of in-hospital mortality (4.8 vs. 3.6%, *p* < 0.001). Tracheostomy use (8.0 vs. 1.4%, *p* < 0.001), pneumonia (15.6 vs. 5.5%, *p* < 0.001), prolonged mechanical ventilation (16.1 vs. 3.5%, *p* < 0.001), reintubation, and 30-day non-elective readmission rates were also increased among patients diagnosed with LC. Those with laryngeal injury also had higher rates of neurologic, cardiac, thrombotic, and infectious complications (Table 2). Patients with LCs experienced longer LOS (15 days [IQR 9–25] vs. 8 [IQR 6–12], *p* < 0.001) and accumulated greater hospitalization costs ($65,200 [IQR 44,800–100,100] vs. 42,700 [IQR 31,800–60,500], *p* < 0.001). In addition, rates of non-home discharge were significantly increased among patients with laryngeal injury.

**Table 2:** Unadjusted clinical outcomes following cardiac operations stratified by laryngeal complications

Parameter	LC (*n* = 39,688)	nLC (*n* = 2,279,940)	*p*-value
Complications (%)			
Neurologic	12.4	2.2	< 0.001
Cardiac	9.4	7.3	< 0.001
Thrombotic	2.8	1.5	< 0.001
Infectious	11.3	6.1	< 0.001
Outcomes (%)			
In-hospital mortality	4.8	3.6	< 0.001
Pneumonia	15.6	5.5	< 0.001
Prolonged ventilation (> 96 h)	16.1	3.5	< 0.001
Reintubation	16.8	5.3	< 0.001
Tracheostomy	8.0	1.4	< 0.001
Non-home discharge	44.4	18.8	< 0.000
30-day non-elective readmission	15.8	11.3	< 0.001
Length of stay (days, median, IQR)	15 [9–25]	8 [6–12]	< 0.001
Cost ($1000s, median, IQR)	65.2 [44.8–100.1]	42.7 [31.8–60.5]	< 0.001

*LC* laryngeal complications, *nLC* no diagnosed laryngeal complications, *SD* standard deviation

On multivariable analysis, several comorbidities were significantly associated with postoperative LC, including coagulopathy, congestive heart failure, and endocarditis (Table 3). Importantly, elective admissions were associated with reduced odds of laryngeal injury (AOR 0.76, 95% CI 0.73–0.79). Notably, patients who underwent multi-valve operations had increased likelihood of developing LC (AOR 1.51, 95% CI 1.36–1.67, ref: isolated CABG). Admission to HVH was associated with reduced odds of LC (AOR 0.58, 95% CI 0.51–0.64), while teaching hospitals were associated with an increased likelihood (AOR 1.20, 95% CI 1.13–1.27).

**Table 3:** Factors associated with post-cardiac surgery laryngeal complications

Parameter	AOR (95% CI)	*p*-value
Age (per year)	1.04 (1.03–1.04)	< 0.001
Elixhauser index (per point)	1.00 (0.99–1.02)	0.28
Female sex (reference: male)	1.08 (1.04–1.12)	< 0.001
Elective admission	0.76 (0.73–0.79)	< 0.001
Operation category		
Isolated CABG	Ref	
Isolated valve	1.30 (1.23–1.38)	< 0.001
CABG + valve	1.41 (1.34–1.49)	< 0.001
Multiple valve	1.51 (1.36–1.67)	< 0.001
Chronic lung disease	0.99 (0.95–1.04)	0.15
Coagulopathy	1.12 (1.07–1.18)	< 0.001
Congestive heart failure	1.14 (1.10–1.19)	< 0.001
Endocarditis	1.34 (1.20–1.50)	< 0.001
End-stage renal disease	1.08 (1.98–1.20)	0.10
Liver disease	1.12 (1.02–1.24)	0.015
Hospital volume		
LVH	Ref	
MVH	0.57 (0.50–0.65)	< 0.001
HVH	0.58 (0.51–0.64)	< 0.001
Teaching hospital	1.20 (1.13–1.27)	< 0.001

*AOR* adjusted odds ratio, *95% CI* 95% confidence interval, *CABG* coronary artery bypass grafting, *LVH* low volume hospital, *MVH* medium volume hospital, *HVH* high volume hospital

**Fig. 2:**
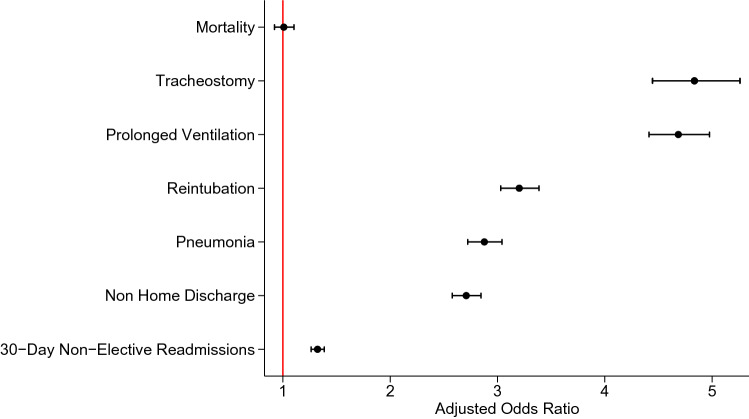
Multivariable risk association of poor perioperative outcomes with patients suffering post-cardiac surgery laryngeal complications (models included adjustment for age, sex, elective admission, Elixhauser Comorbidity Index, endocarditis, congestive heart failure, end stage renal disease, coagulopathy, liver disease, chronic lung disorder, teaching hospital, hospital cardiac surgery volume and operative category)

**Fig. 3:**
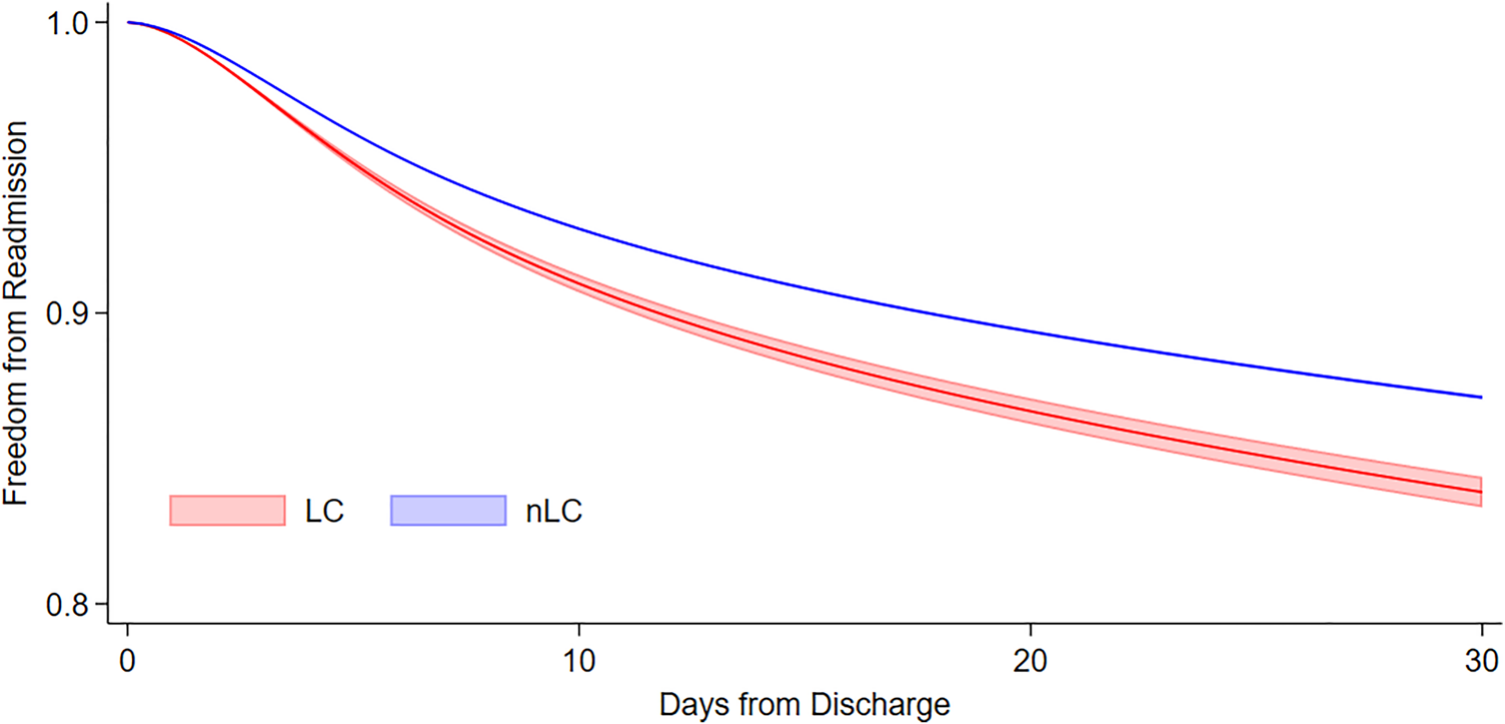
Standardized effect of laryngeal complications (LC) on non-elective readmissions following cardiac surgery derived using Royston–Parmer flexible parametric model. *nLC* no laryngeal complications

Despite no association of LC with in-hospital mortality (AOR 1.01, 95% CI 0.92–1.10, Supplemental Table 2), increased odds of pneumonia, postoperative tracheostomy, prolonged mechanical ventilation, and reintubation were observed (Fig. 2). Laryngeal complications were associated with a 7.7-day increment in LOS (95% CI 7.4–8.0 days) and $24,200 (95% CI $23,000–25,400) increase in attributable hospitalization costs. Additionally, LC was associated with increased likelihood of non-elective, 30-day readmission (AOR 1.32, 95% CI 1.26–1.39), and non-home discharge (AOR 2.71, 95% CI 2.58–2.85). On Royston–Parmar survival analysis, LC demonstrated increased risk of readmissions within 30 days (Fig. 3).

## Discussion

In this population-based study of cardiac surgical patients, we report a rise in the diagnosis of LCs over the past decade. Factors including age, female sex, operative complexity, and hospital-level characteristics were found to be independently associated with LC. While not associated with in-hospital mortality, laryngeal injury was associated with increased perioperative complications, resource utilization, and 30-day, non-elective readmissions. These findings warrant further discussion.

The reported incidence of LCs among cardiac surgical patients varies widely, ranging from 7 to 16% in single-center studies [3, 7, 10, 28]. Using a large cohort, we report an incidence of 1.7% which is concordant with a similar study of non-cardiac, thoracic surgery patients [5]. The higher rates of laryngeal injury observed in institutional series are likely attributable to the methodological differences between prospective and retrospective analyses, as well as the occult presentation and lack of standardized screening measures for LC. To our knowledge, the present study is the first to demonstrate the rising incidence of LCs following cardiac operations. While this finding may reflect more accurate coding in recent years, the continued rise of diagnoses is more likely explained by the gradual adoption of objective, diagnostic evaluations aimed to actively identify postoperative laryngeal conditions. Undoubtedly, uniform screening protocols are necessary to capture the true incidence of these LCs in this high-risk population.

The present study found several perioperative factors to be associated with greater odds of laryngeal injury, including advanced age, female sex, and increasing operative complexity. Prior literature of non-surgical patients attributes the increased prevalence of dysphagia among women to heightened bronchial sensitivity and smaller airway size, although further studies are necessary to delineate contributing factors [29]. Multi-valve procedures may be associated with increased risk of LC due to prolonged operative time and complexity. Our previous institutional analysis demonstrated operative time to be associated with the development of dysphagia in a cohort of liver transplant recipients [11]. Several reasons including severity of underlying disease, duration of intubation, and additional TEE utilization for valve evaluation may account for the increased risk of LC associated with more complex operations. Specifically, extensive use of TEE in valvular operations may result in compression of the pharyngoesophagus and possibly transmit compression of the laryngeal nerve. Further investigation to define the role of TEE use in the development of LC is warranted.

Our analysis also identified certain hospital-level factors to be associated with LCs. Operations at teaching hospitals were independently associated with LC, a finding that may simply represent an identification bias reflecting increased diagnostic capabilities and complexity of surgical cases at such facilities [30, 31]. Other inherent considerations may potentially contribute to this observation, namely the impact of trainees performing endotracheal intubations and prolonged TEE examinations. In fact, a prospective multicenter study conducted by Sanders and colleagues demonstrated increased risk of intubation-associated events with trainee participation [32]. At the hospital level, admissions to higher volume cardiac surgical centers demonstrated a protective effect against laryngeal injury. Low volume centers have previously been linked to inferior outcomes for complex procedures across several surgical specialties [33–35]. These hospitals may have less experienced staff and fewer resources including dedicated critical care services [36]. The observed variation in LC incidence across hospital types highlights the presence of opportunity for quality improvement and mitigation of LCs through best practices such as rapid extubation and reduction in operative times.

In congruence with several institutional series, we found no significant association between laryngeal injury and mortality [2, 37]. An important finding of the present work is the year-over-year reduction in mortality among patients with LC. Increased recognition, tools for definitive diagnosis, and advancements in standards of management for subsequent complications may be responsible for this phenomenon.

Perioperative complications, resource use, and non-elective readmissions were greater among patients diagnosed with laryngeal injuries. Similarly, Crowson et al. reported limitations of vocal fold motion to be associated with greater odds of pneumonia and tracheostomy among general thoracic surgery patients [5]. In addition to airway protection during swallowing, vocal fold dysfunction impairs the ability to cough and clear secretions. While the NRD does not provide granularity that may elucidate the mechanisms related to perioperative complications, it is probable that the majority of patients with LC developed postoperative pneumonia secondary to silent aspiration. The presented data demonstrate significant associations between laryngeal injury and poor hospitalization course, suggesting that the development of targeted mitigation strategies is necessary to improve clinical and financial outcomes following cardiac surgery.

To date, several reports have suggested a more proactive approach to in-hospital laryngeal evaluations. Hinchey et al. reported that adherence to strict screening protocols in stroke patients significantly reduced the incidence of aspiration pneumonia [38]. A priori identification of patients at risk for developing poor laryngeal function remains ill-defined with some groups having suggested a risk-stratification scheme based on patient and operative factors. Nonetheless, risk indices such as the risk of dysphagia in cardiac surgery (RODICS) score are not widely utilized and require further validation in larger cohorts [1]. Our group previously validated the use of a 3-phase bedside swallow screen and FEES in diagnosing dysphagia among liver transplant recipients [11]. Although bedside swallowing evaluations could be used as non-invasive first line screening tools, their pragmatic applicability and sensitivity remain to be seen in cardiac surgical populations. Further studies regarding the potential benefit of early detection for laryngeal injury following cardiac surgery are warranted.

Our study has several important limitations. Due to the retrospective nature of our study, we were only able to infer correlation and not causation regarding risk factors for LC and the associated outcomes. The lack of standardized screening protocols and occult presentation may contribute to underreporting of LC in the NRD. Consequently, patients who were identified as without LC may have been wrongly coded as such. Furthermore, ICD coding fails to provide information regarding the timing of diagnosis, thus, preventing the identification of patients who had preoperative voice or swallowing disturbances. Clinical variables such as operative time, intraoperative events, use of TEE, and modalities used for diagnoses were not available for analysis. Notwithstanding the inherent shortcomings of the chosen study design, the advantages are held in that the present analysis utilizes the largest, all-payer dataset to characterize LCs, and their impact on outcomes and costs at a national level, allowing for greater generalizability of our findings.

## Conclusions

In this retrospective national study, we identified several risk factors for LCs following cardiac surgery including female sex, increased operative complexity and management at teaching hospitals. While laryngeal injuries were not associated with overall mortality, the presence of these diagnoses were associated with greater complication rates, resource use, and readmission. Further identification of factors associated with this important subgroup of complications may help guide the implementation of routine, postoperative screening protocols, increase early detection, and ultimately yield improved outcomes.

## Supplementary Information

Below is the link to the electronic supplementary material.

Supplementary file1 (PDF 16 kb)
