# Supplementary material for: Incidence and Outcomes of Laryngeal Complications Following Adult Cardiac Surgery: A National Analysis
Source: Dysphagia. 2021 Oct 21;37(5):1142–50. doi: 10.1007/s00455-021-10377-2 (PMC9463246; doi:10.1007/s00455-021-10377-2)
Supplement: Supplementary file 1 — Supplementary file1 (PDF 16 kb) [file 455_2021_10377_MOESM1_ESM.pdf]

Supplemental Material

Supplemental Table 1: ICD 9 and ICD 10 codes

Diagnosis	ICD-9 Diagnosis Code	ICD-10 Diagnosis Code
Vocal Fold Paralysis	478.3x	J3800, J3801, J3802
Dysphagia/Aphagia	787.2x	R131, R13, R633
Dysphonia/Aphonia	784.4x	R490, R498, R499

Supplemental Table 2: Factors associated with mortality among those undergoing cardiac operations, including vocal fold dysfunction.

Parameter	AOR (95% CI)	P-Value
Vocal Fold Dysfunction	1.01 (0.92, 1.10)	0.86
Female (reference: Male)	1.42 (1.38-1.46)	<0.001
Age (per year)	1.04 (1.04-1.04)	<0.001
Elixhauser Index (per point)	1.02 (1.01-1.03)	<0.001
Elective Admission	0.58 (0.56-0.60)	<0.001
Operation Category		
Isolated CABG	Ref	
Isolated Valve	1.35 (1.30-1.41)	<0.001
CABG+Valve	2.15 (2.07-2.23)	<0.001
Multiple Valve	2.32 (2.18-2.47)	<0.001
Congestive Heart Failure	1.70 (1.65-1.76)	<0.001
Chronic Lung Disease	1.01 (0.97-1.04)	0.64
End Stage Renal Disease	3.16 (3.02-3.31)	<0.001
Liver Disease	7.00 (6.70-7.32)	<0.001
Coagulopathy	1.26 (1.22-1.31)	<0.001
Endocarditis	2.20 (2.06-2.36)	<0.001
Hospital Volume		
LVH	Ref	
MVH	0.74 (0.67-0.81)	<0.001
HVH	0.62 (0.56-0.68)	<0.001
Teaching Hospital	0.93 (0.89-0.98)	0.002

(*AOR: Adjusted Odds Ratio; 95% CI: 95% Confidence Interval; CABG: Coronary Artery Bypass Grafting; LVH: Low Volume Hospital; MVH: Medium Volume Hospital; HVH: High Volume Hospital)*
